# Hepato-protective effects and chemical constituents of a bioactive fraction of the traditional compound medicine-Gurigumu-7

**DOI:** 10.1186/s12906-016-1156-3

**Published:** 2016-06-13

**Authors:** Haiyan Xu, Qiong Ma, Jiannan Ma, Zhigang Wu, Yali Wang, Chaomei Ma

**Affiliations:** Collage of Life Science, Inner Mongolia University, Huhhot, Inner Mongolia China

**Keywords:** Gurigumu-7, Hepatoprotective effect, Bioactive fraction, Bioactive constituents

## Abstract

**Background:**

Gurigumu-7 is an important traditional Mongolian medicine frequently used for liver diseases. However, the pharmacological effects and the bioactive constituents are not well understood.

**Method:**

This research was to use CCl_4_-induced liver damage in mice to investigate the hepatoprotective effects of Gurigumu-7 and the methanol eluted fraction from a DIAION column of an extract of Gurigumu-7 (MF). The chemical constituents of MF were analyzed by UPLC-MS.

**Results:**

Pretreated orally with MF (66, 132 and 264 mg/kg) once a day for 4 days dose-dependently suppressed CCl_4_-induced mice liver histopathological changes and serum aminotransferase activities (alanine transaminase: 1144.0 ± 787.2 *v.s*. 2461.8 ± 1072.7 U/L, *p* < 0.05; aspartate transaminase: 1173 ± 785.3 *v.s.* 2506.6 ± 1140.7 U/L, *p* < 0.01). MF treated group demonstrated increased levels of SOD (108.19 ± 30.32 *v.s.* 75.75 ± 5.37 U/mg protein, *p* < 0.01) but decreased levels of malonyldialdehyde (7.68 ± 1.95 *v.s.* 44.32 ± 16.68 nmol/mg protein, *p* < 0.01) compared to the CCl_4_ control group. More than 30 chemical constituents were quantified, and MF was found to be rich in ellagic acid (297.97 mg/g), luteolin and its glucosides (35.10 mg/g), apigenin and its glucosides (>30 mg/g), ursolic acid (14.91 mg/g), bidenoside C (8.75 mg/g), and proanthocyanidins (15.64 mg/g in proanthocyanidin A2 equivalent).

**Conclusion:**

The methanol eluted fraction (MF) from a DIAION column of an extract of the Mongolian medicine-Gurigumu-7 was found to be more hepatoprotective than Gurigumu-7. The results suggested that MF is a promising bioactive fraction for the development of new hepatoprotective medicine with better formulation and quality control properties.

## Background

Gurigumu-7 is an ethnic compound medicine frequently used for liver diseases in the Mongolian and Tibetan traditional medical settings. It is comprised of seven individual traditional medicines, the flower of *Carthamus tinctorius* L. (Safflower), the fruit of *Terminalia chebula* Retz. (Fructus chebulae), the flower of *Scabiosa comosa* Fisch. ex Roem. & Schult. (Flos scabiosae), the aerial part of *Ephedra sinica* Stapf (Herba ephedrae), the aerial part of *Viola yezoensis* Maxim. (Herba violae), gypsum and the caulis of *Clematis armandii* Franch (Caulis clematidis armandii).

Safflower is one of the most frequently used herbal drugs found in traditional medicine prescriptions for its function to improve circulation and lower blood pressure [1, 2]. The major flavonoid constituents of safflower were reported to have hepatoprotective effects on CCl_4_-induced liver injury [3]. Fructus chebulae has been used in traditional medicine for intestinal and hepatic detoxification, diarrhea, cough, sore throat and various ailments [4]. Fructus chebulae is rich in phenolic compounds which were reported to have anti-viral activities against hepatitis C and other viruses [5–7]. Flos scabiosae is used mainly for liver diseases in traditional medicine [8]. Phenolics [9, 10] and triterpene compounds [11] were reported as the bioactive constituents of Flos scabiosae. Herba ephedrae has been used to relief symptoms of colds [12] due to the nasal decongesting and bronchodilating effects of its alkaloid constituents, ephedrine and related compounds. However, these sympathomimetic alkaloids may cause side effects to central nervous system. Renewed interest in herba ephedrae has come from new pharmacological findings that this herb could decrease uraemic toxins and showed anti-inflammatory activity due to its proanthocyanidin constituents [13–16]. Herba violae has been used for boils, carbuncles, hepatitis and other infections in traditional Chinese medicine [17]. The flavonoid constituents were reported to be the bioactive constituents of Herba violae for antibacterial and antioxidant activities [18–20]. Caulis clematidis armandii has been traditionally used mainly for inflammatory-associated diseases and the phenolic constituents were reported to be its bioactive constituents [21, 22]. Gurigumu-7 was reported have the effect to decrease serum aminotransferase activities in CCl_4_-induced mice [23]. However, the bioactive fraction, bioactive constituents and mechanism of action of Gurigumu-7 have not been revealed.

Composed of 7 raw materials, Gurigumu-7 has to be administered in large volumes and the taste is unpleasant. Moreover, it is hard to establish a quality control method for Gurigumu-7, as each of the component herbal medicine could contain hundreds of chemical constituents. It is necessary to find out the bioactive fraction for the possibility to reduce the dosage and to simplify the quality control method. In the present study, we used macroporous resin to separate Gurigumu-7 extract to 3 fractions and tested the hepatoprotective effects of these fractions. The most active fraction, MF (methanol eluted fraction), was further investigated in detail for its in vivo protective effects on liver damage induced by carbon tetrachloride and compared the effect with Gurigumu-7. The chemical constituents, antioxidant activity and ability to increase liver antioxidase load of MF were also investigated.

## Methods

### Chemicals and instruments

Extraction solvents were of analytical grade from XiLong chemical Co. Ltd. (Guangdong, China). Silymarin was obtained from Sigma-Aldrich (SIGMA-ALDRICH, Co., China) and used as a positive control in this research. UPLC-DAD-ESI-MS experiments were performed on an Agilent 1290 infinity UPLC system (Agilent, USA). Absorbance was measured with a microplate reader (DNM-9602, Beijing Pu Long new technology Co. Ltd., Beijing, China). A wan-neng pulverizer (Zhejiang Yi Li Co. Ltd., Zhejiang, China) was used for grinding medicines.

### Plant material

The seven medicines to formulate Gurigumu-7 in this research were supplied by Kulun Mongolian medicine factory, Inner Mongolia, China and identified by the authors through examining the morphological characteristics, anlyzing the UHPLC-MS of the plant extracts, and considering the information provided by the supplier. The voucher specimens were stored in the Laboratory of Natural Products & Functional Foods, College of Life Sciences, Inner Mongolia University, China, as followings: the flower of *Carthamus tinctorius* L (voucher specimen number NPFFC-2); the fruit of *Terminalia chebula* Retz (voucher specimen NPFFT-1); Gypsum (99.5 % of CaSO_4_•2H_2_O content as determined by the method described in Chinese pharmacopeia, voucher specimen number NPFFG-1); the aerial part of *Ephedra sinica* Stapf (voucher specimen number NPFFE-1); the aerial part of *Viola yezoensis* Maxim (voucher specimen number NPFFV-1); the flower of *Scabiosa comosa* Fisch. ex Roem. & Schult. (voucher specimen number NPFFS-1); the caulis of *Clematis armandii* Franch (voucher specimen number NPFFC-3).

### Preparation of samples

Gurigumu-7 was prepared according to the documented prescription [1] by mixing the individual medicines in the following ratio: Safflower 25 g – Gypsum 15 g – Herba ephedrae 15 g – Herba violae 15 g – Fructus chebulae 15 g – Flos scabiosae 10 g – Caulis clematidis armandii 10 g, and grinding the mixture to powder.

MF (methanol eluted fraction from a DIAION column of Gurigumu-7 extract) was prepared as following: Gurigumu-7 was extracted with methanol under reflux (70 °C) for three times (2 h, 1 h and 30 min, respectively). The pooled methanol solution was concentrated under vacuum (40 °C) to get the methanol extract (10.33 g) which was subjected to a macroporous resin (DIAION HP20) column eluted with H_2_O, H_2_O–MeOH 1:1 and MeOH. The MeOH eluted part was concentrated under vacuum (40 °C) to get MF as a brown powder (1.78 g).

### Animals and treatments

Six-week-old male scxk (meng) 2002–0001 mice (weighing 30 ± 5 g) were purchased from the Animal Center of Inner Mongolia University, China. The mice were housed in clean cages accessing to food and water ad libitum and acclimated to the temperature (22 ± 2 °C) with 12 h light/dark cycles for one week. The animals were cared for in accordance with the “guidelines for animal experiments” and the experimental procedures were approved by the Animal Ethics Committee of Inner Mongolia University (approval number 2016004). One hundred and twenty mice were randomly divided into two groups named experiment 1 and 2 (e1 and e2). E1 and e2 were further randomly divided into six groups with ten mice in each group, respectively. Mice of groups 1 and 2 from e1 and e2 were given with 0.5 ml saline/day, group 3 from e1 and e2 were given with 17 mg/kg silymarin. Groups 4, 5 and 6 from e1 were treated with Gurigumu-7 powder (270.84, 541.68, 1083.36 mg/kg, respectively). Groups 4, 5 and 6 from e2 were treated with 66, 132 and 264 mg/kg of MF, respectively. Each group received the appropriate vehicle or sample daily by gastric intubations for 4 days. After 1 h of the medication in the forth day, CCl_4_ (125 *μ*l, 1 % in oil) was given by intraperitoneal injection to mice except for the mice in group 1 which were given oil only. Whole blood was collected for biochemical analysis from the orbit 16 h later after drug administration, and the liver tissue was cut and immediately fixed in 10 % neutral formalin for histopathology study.

### Serum biochemistry

The blood samples were put standing for one hour and the serum was separated by centrifugation (WiseSpin®Personal Table Top Centrifuges, CF-10, DAIHAN Scientific, Co., Ltd) under 12225 g for 5 min. Serum alanine transaminase (ALT) and aspartate transaminase (AST) were measured in Hospital of Inner Mongolia University using standard clinical method.

### Determination of malondialdehyde (MDA) and superoxide dismutase (SOD) in liver homogenate

The liver tissue samples were homogenized with cold saline. The homogenates were centrifuged at 3000 rpm at 4 °C for 10 min and the supernatant was kept at −80 °C until use. The supernatant was used for the measurement of MDA and SOD using commercial kits (Jiancheng Institute of Biotechnology, Nanjing, China) following the supplier’s instructions.

### Histological examinations

Liver tissues from e2 were fixed in 10 % (v/v) neutral phosphate buffer formalin and the liver pathological section were prepared and examined by the First Affiliated Hospital of Inner Mongolia Medical University – Cancer Hospital.

### DPPH radical scavenging assay

The DPPH scavenging activity of MF and the Gurigumu-7 extract were measured at the concentrations of 50, 25, 12.5, 6.25, 3.125 *μ*g/mL using reported method [24]. Briefly, 10 μl of sample solution in DMSO was mixed with 190 μl of the ethanol solution of DPPH. After 20 min, the absorbance (A) at 540 nm was measured and the percentage of DPPH scavenged (S%) was calculated using the following formula:

$$ \mathrm{S}\%=100\times \left({\mathrm{A}}_{\mathrm{control}}\hbox{-} {\mathrm{A}}_{\mathrm{sample}}\right)/{\mathrm{A}}_{\mathrm{control}} $$

Where A_control_ was the average absorbance of wells without sample.

Results represented as EC_50_ (sample concentration that produced 50 % of radical scavenging activity) were found from the S%-versus-concentration curves.

### Preparation of Sample Solutions for UPLC-DAD-MS Analysis

The 3 fractions of Gurigumu-7 were dissolved in DMSO (10 *μ*g:1 ml) containing 1 *μ*g/mL of abrusin 2″-*O-β*-apioside [25] as internal standard. Standard stock solutions were prepared in DMSO containing 1 *μ*g/mL of internal standard. The solutions were filtrated through 0.22 *μ*m microfilters to obtain the sample solutions for ultra-high performance liquid chromatography-diode array detector-triple quadruple mass spectrometry (UPLC-DAD-QQQMS) analysis.

### UPLC-DAD-QQQMS analysis

The chemical compositions of the three fractions from a DIAION column chromatography of Gurigumu-7 were analyzed by UPLC-DAD-QQQMS using an Agilent ZORBAX SB-C18 RRHT column (50 × 2.1 mm i.d.; particle size 1.8 μm) at 30 °C. The constituents were quantified by UHPLC-QQQMS in multiple reaction monitoring (MRM) mode using reported analysis conditions and standard compounds for the constituents of Safflower [26, 27], Fructus chebulae [28], Flos scabiosae [10], Herba ephedrae [29], and Herba violae [20, 30]. For the quantification of calceorioside B in Caulis clematidis armandii, ESIMS full scan method was used and the extract was compared with a standard compound from Beijing Century Aoke Biotechnology Co. Ltd (Beijing, China). Data were presented as the average values from three repeat quantifications.

### Measurement of proanthocyanidin A2 equivalents

Proanthocyanidin A2 equivalents were measured by n-BuOH–HCl–Fe III method [31, 32]. Briefly, to a 1.5 ml centrifuge tube was added 0.5 mg of sample, 1.2 ml of a solution of n-BuOH-conc. HCl (95:5, v/v) and 40 *μ*l of ferric ammonium sulphate reagent (2 % w/v in 2 M HCl). The tubes were closely capped with top clips and heated at 95 °C for 40 min. Absorbance of the released cyanidin was measured at 560 nm by a Thermo Scientific Varioskan Flash (Thermo Fisher Scientific Oy D.O. Box100, FI-01621 Vantaa, Finland). The flavan-3-ol contents were expressed as proanthocyanidin A2 equivalents as A-type proanthocyanidins were reported from one of the medicines in Gurigumu-7, Herba ephedrae [15, 16]. Proanthocyanidin A2 equivalents of samples were calculated from the absorbance – concentration curve of proanthocyanidin A2.

### Statistical analysis

Statistical analysis of the data was accomplished by mean of the SPSS® statistical software package. The data are presented as the means ± SD. Differences where *P* < 0.05 were considered statistically significant.

## Results

### The effect of gurigumu-7 and MF on the increased levels of serum transaminases induced with CCl_4_

As shown in Tables 1 and 2, the serum ALT and AST levels of CCl_4_-treated groups were significantly higher (*P* < 0.01) than saline-treated groups. In e1, the serum ALT and AST levels of mice treated with silymarin were lower (*P* < 0.05) than those treated with CCl_4_ only. Pretreatment with 1083.36 mg · kg^−1^ of Gurigumu-7 powder significantly decreased the AST level (*p* <0.05). Gurigumu-7 was separated by DIAION column chromatography to obtain three fractions and the methanol eluted fraction, MF, was found to be the most effective fraction in reducing serum transaminases, in a preliminary animal experiment. MF was then investigated in detail in e2 and the results are shown in Table 2. Pre-administration of MF to mice for four days at 66, 132 and 264 mg/kg reduced the elevation of serum ALT and AST levels dose dependently. The serum ALT and AST levels in the 264 mg/kg MF-treated group were significantly lower (*p* <0.05 and *p* <0.01, respectively) than CCl_4_-treated group.

**Table 1:** Effects of Gurigumu-7 powder on serum transaminases in CCl_4_-injured mice (e1)

Groups	Parameter (U/L)	
	ALT	AST
Saline-treated mice	32.1 ± 8.4^##^	141.6 ± 59.0^##^
CCl_4_-treated mice	3822.0 ± 1494.8	4059.6 ± 1408.3
CCl_4_-17 mg/kg silymarin-treated mice	2205.0 ± 912.2*	2034.0 ± 1262.9*
CCl_4_-270.84 mg/kg Gurigumu-7 -treated mice	3438.0 ± 1742.0	3586.0 ± 2170.1
CCl_4_-541.68 mg/kg Gurigumu-7-treated mice	2933.2 ± 1405.0	2910.0 ± 1652.8
CCl_4_-1083.36 mg/kg Gurigumu-7 -treated mice	2345.1 ± 1449.8	2290.2 ± 1519.7*

The values are expressed as mean ± S.D

*significant different (*p* <0.05) from CCl_4_ control group

^##^highly significant different (*p* <0.01) from CCl_4_ control group

**Table 2:** Effects of MF on serum transaminases in CCl_4_-injured mice (e2)

Groups	Parameter (U/L)	
	ALT	AST
Saline-treated mice	30.4 ± 4.0^##^	123.0 ± 24.3^##^
CCl_4_-treated mice	2461.8 ± 1072.7	2506.6 ± 1140.7
CCl_4_-17 mg/kg silymarin-treated mice	1115.6 ± 291.3**	1230.6 ± 437.6**
CCl_4_-66 mg/kg MF-treated mice	2766.0 ± 1909.7	2482.8 ± 1853.3
CCl_4_-132 mg/kg MF-treated mice	1929.0 ± 799.8	1748.0 ± 694.8
CCl_4_-264 mg/kg MF-treated mice	1144.0 ± 787.2*	1173 ± 785.3**

The values are expressed as mean ± S.D

*significant different (*p* <0.05) from CCl_4_ control group

^##,^**highly significant different (*p* <0.01) from CCl_4_ control group

### Effects on Superoxide Dismutase (SOD) and Malondialdehyde (MDA) levels

In order to evaluate the effects of MF on CCl_4_ induced liver oxidative stress, we examined the mouse liver levels of SOD and MDA. SOD is an important antioxidant enzyme produced by living organisms to defense oxidative stress [33]. MDA is produced by lipid peroxidation and its level correlates with the degree of oxidative stress. The liver SOD and MDA levels are shown in Table 3. CCl_4_ treatment significantly decreased SOD activity and increased MDA content (*P* < 0.01), suggesting strong oxidative stress and lipid peroxidation in the CCl_4_ treated group. Pretreatment with MF (66, 132 and 264 mg/kg) and silymarin prevented this trend. The SOD levels in both silymarin treated group and MF treated groups (264 mg/kg) were significantly increased compared with the CCl_4_ control group, and the MF treated group have better activities. Pre-administration with silymarin and MF caused highly significant decreases in the liver level of MDA (*P* < 0.01).

**Table 3:** Liver SOD and MDA levels in mouse treated with CCl_4_ and MF

Groups	SOD (U/mg protein)	MDA (nmol/mg protein)
Saline-treated mice	114.23 ± 2.75^##^	7.08 ± 0.04^##^
CCl_4_-treated mice	75.75 ± 5.37	44.32 ± 16.68
CCl_4_-17 mg/kg silymarin treated mice	102.41 ± 24.66^*^	12.47 ± 3.27^**^
CCl_4_-66 mg/kg MF treated mice	82.46 ± 15.48	19.31 ± 6.58^**^
CCl_4_-132 mg/kg MF treated mice	93.39 ± 40.91	8.58 ± 1.11^**^
CCl_4_-264 mg/kg MF treated mice	108.19 ± 30.32^**^	7.68 ± 1.95^**^

The values are expressed as mean ± S.D

*significant different (*p* <0.05) from CCl_4_ control group

^##,^**highly significant different (*p* <0.01) from CCl_4_ control group

### Histopathological changes of mice livers

**Fig. 1:**
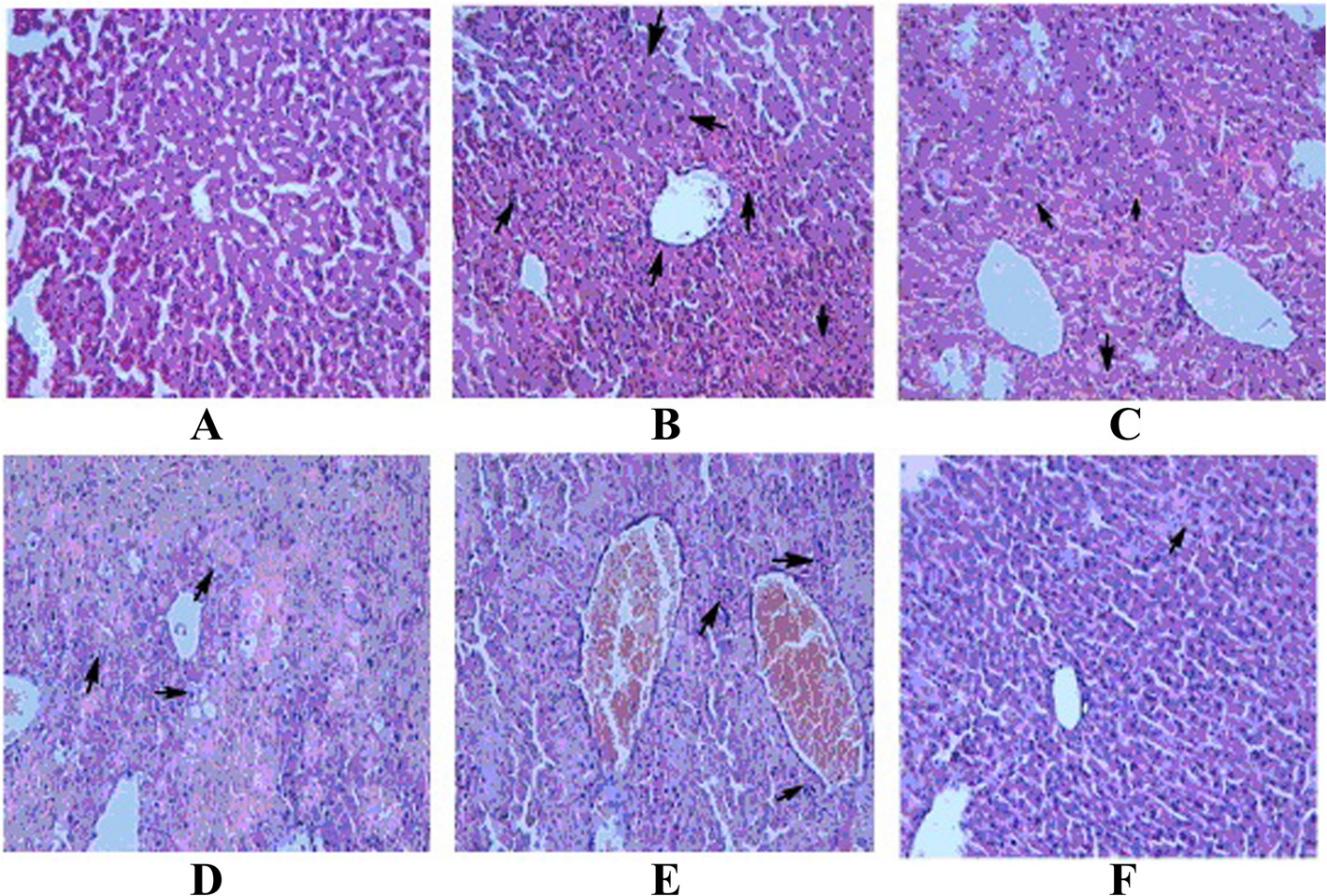
Effects of silymarin and different doses of MF on the liver histological changes in CCl_4_ treatment mice. **a** normal group; **b** CCl_4_-intoxicated group; **c** CCl_4_ + Silymarin (17 mg/kg); **d** CCl_4_+ MF (66 mg/kg); **e** CCl_4_+ MF (132 mg/kg). **f** CCl_4_+ MF (264 mg/kg)

The microscopic pictures of the mice liver tissue section are shown in Fig. 1. The cells of normal group (group 1, Fig. 1a) were arranged regularly without obvious degeneration and necrosis. In CCl_4_-intoxicated group (group 2, Fig. 1b), the liver lobule was around the central vein and there was a wide range of focal necrosis with inflammatory cell and degeneration. In the positive control group (group 3, Fig. 1c), the cells were cloudy swelling, but the structure was intact. As the dose of MF increased (Fig. 1d, e, f), the morphological changes became less.

### Components of MF

More than 30 chemical constituents were quantified by UHPLC-QQQMS and colorimetric method for MF and the other two fractions from the DIAION column. The results are listed in Table 4. MF was found to be especially rich in ellagic acid (297.97 mg/g), luteolin and its glucosides (35.10 mg/g), apigenin and its glucosides (30.07 mg/g), ursolic acid (14.91 mg/g), and bidenoside C (8.75 mg/g). Among these, the triterpene compound ursolic acid (a major constituent of Flos scabiosae) and the acetylenic compound bidenoside C (a constituent of Safflower) were found exclusively in MF. MF also contained large amount of proanthocyanidins (15.64 mg/g in proanthocyanidin A2 equivalent) that may come from Herba ephedrae, a plant known to contain A-type proanthocyanidins [16]. It is interesting to note that almost all ephedra alkaloids (98.33 %) were eluted out to the H_2_O-MeOH 1:1 fraction, leaving MF almost free of ephedrine and related alkaloids.

**Table 4:** Concentrations (mg/g unless otherwise indicated) of Gurigumu-7 constituents in the three fractions

Main source	constituents	H_2_O fraction	1:1 fraction	MF
Safflower	protocatechuic acid	0.00 ± 0.00	1.61 ± 0.01	1.15 ± 0.00
	hydroxysafflor yellow A	nd	12.77 ± 0.01	0.19 ± 0.02
	6-hydroxykaempfer 3,6,7-triglucoside	0.17 ± 0.00	2.09 ± 0.00	0.08 ± 0.01
	kaempferol-3-*O*-D-glucoside	nd	0.082 ± 0.04	3.13 ± 0.01
	bidenoside C	nd	nd	8.75 ± 0.01
	kaempferol-3-*O*-rutinocoside	0.06 ± 0.01	0.65 ± 0.05	2.86 ± 0.04
	6-hydroxykaempferol-3-rutinoside-6-glucoside	nd	1.63 ± 0.02	0.22 ± 0.02
	6-hydroxykaempferol-3,6-diglucoside	nd	0.28 ± 0.039	0.04 ± 0.03
	linolic acid	nd	0.21 ± 0.03	26.2 ± 0.09
	*α*-linolenic acid	nd	0.20 ± 0.08	25.5 ± 0.13
	oleic acid	0.36 ± 0.07	0.38 ± 0.10	3.13 ± 0.09
Fructus chebulae	chebulic acid	12.81 ± 0.02	0.74 ± 0.00	0.076 ± 0.00
	gallic acid	7.68 ± 0.02	12.23 ± 0.02	nd
	chebumeinin A	0.09 ± 0.00	0.67 ± 0.01	0.09 ± 0.00
	chebumeinin B	0.24 ± 0.01	0.48 ± 0.01	0.24 ± 0.01
	casuarinin	nd	2.35 ± 0.01	1.88 ± 0.01
	corilagin	3.48 ± 0.01	7.30 ± 0.02	3.50 ± 0.02
	chebulagic acid	2.25 ± 0.01	6.05 ± 0.01	2.38 ± 0.01
	pentagalloyl glucose	0.86 ± 0.01	1.06 ± 0.01	0.94 ± 0.02
	ellagic acid	nd	82.14 ± 0.80	297.97 ± 1.84
Flos scabiosae	caffeic acid	nd	0.46 ± 0.00	nd
	quinic acid	nd	2.28 ± 0.01	nd
	chlorogenic acid	0.87 ± 0.01	32.08 ± 0.58	0.83 ± 0.02
	*p*-coumaric acid	0.24 ± 0.00	0.34 ± 0.00	0.67 ± 0.01
	luteolin-6-*C*-glucoside	nd	5.54 ± 0.01	0.64 ± 0.01
	quercetin-3-glucoside	nd	0.98 ± 0.01	4.30 ± 0.01
	rutin	nd	nd	0.05 ± 0.00
	apigenin-7-arabinoglucoside	nd	0.75 ± 0.00	5.38 ± 0.02
	apigenin-4′-glucoside	nd	nd	11.59 ± 0.02
	apigenin-7-glucoside	nd	nd	11.51 ± 0.01
	luteolin-4′-*O*-glucoside	nd	0.73 ± 0.00	16.02 ± 0.02
	luteolin-7-*O*-glucoside	nd	0.84 ± 0.01	16.12 ± 0.02
	luteolin	nd	nd	2.96 ± 0.02
	apigenin	nd	nd	1.59 ± 0.01
	ursolic acid	nd	nd	14.91 ± 0.35
	hederagenin/3β,23-dihydroxyursan-12-en-28-oic acid	0.01 ± 0.019	0.01 ± 0.02	1.80 ± 0.03
Herba ephedrae	Ephadra alkaloids	nd	98.33 ± 0.15 %^a^	1.67 ± 0.64 %^a^
	Proanthocyanidin A2 equivalent	nd	31.33 ± 2.62	15.64 ± 1.78
Herba violae	6,7-dihydroxycoumarin	nd	25.23 ± 0.74	3.12 ± 0.02
	5,5′-bi (6,7-dihydroxycoumarin)	nd	0.67 ± 0.01	nd
	apigenin 6,8-di-C-*β*-D-glucoside,	0.54 ± 0.53 %^a^	90.81 ± 1.01 %^a^	8.65 ± 0.87 %^a^
	apigenin 6-C-*β*-D-glucosyl-8-C- *α*-L-arabinoside/apigenin 6-C-*β*- D- glucosyl-8-C-*β*-L-arabinoside	2.88 ± 0.36 %^a^	48.96 ± 0.08 %^a^	48.16 ± 0.65 %^a^
	apigenin 6-C-*α*-L-arabinosyl- 8-C-*β*-D-xyloside/apigenin 6,8-di-C-*α*-L-arabinoside	2.66 ± 0.08 %^a^	47.50 ± 0.45 %^a^	49.83 ± 0.71 %^a^
Caulis clematidis armandii	calceolarioside B		0.18 ± 0.89	0.17 ± 0.07

*nd* not detectable

^a^percentages of these compounds were calculated according to the peak area ratios of the compounds in LC-MS

**Table 5:** DPPH scavenging activity of a methanolic extract of Gurigumu-7 and MF

Substance	percentage of DPPH scavenged at different concentration					EC_50_ (μg/mL)
	3.125 μg/mL	6.25 μg/mL	12.5 μg/mL	25 μg/mL	50 μg/mL	
methanolic extract of Gurigumu-7	4.2	10.1	20.6	31.6	55.4	43.7
MF	8.0	9.4	17.9	38.8	59.3	39.8
protocatechuic acid (positive control)	30.4	44.4	58.7	72.2	81.7	7.8

MF is the methanol eluted fraction from a DIAION column of Gurigumu-7 extract

MF demonstrated stronger DPPH scavenging activity (EC_50_ = 39.79 *μ*g/mL) than Gurigumu-7 extract (EC_50_ = 43.69 *μ*g/mL), which may come from the high contents of phenolic compounds such as ellagic acid and the flavonoids, luteolin and its glucosides, as well as apigenin and its glucosides in MF (Table 5).

## Discussion

The ethnic compound medicine, Gurigumu-7, has a long history to be used for liver diseases in Mongolian and Tibetan medical clinics [1]. The formulations of Gurigumu-7 on the market are bitter and astringent powder or bolus with a large dosage indicated (5 g every time for example). They are unpleasant to swallow and the administration may affect appetites. In this study, we separated Gurigumu-7 extract into three fractions by DIAION column and found MF, the methanol eluted fraction, demonstrated better bioactivity than Gurigumu-7. From 10.33 g of Gurigumu-7 extract, 1.78 g of MF was obtained, and indeed, a much lower dose (264 mg/kg) of MF showed more potent hepato-protective activity than Gurigumu-7 at 1083.36 mg/kg. These results indicated that MF is one of the hepato-protective fractions of gurigumu-7, and that administration of MF could reduce the effective dose.

It is reported that liver tissue in CCl_4_ treated animals can cause lipid peroxidation and trigger production of MDA. Measurement of MDA levels is the most commonly used method for the evaluation of lipid peroxidation, because MDA is the most abundant individual aldehyde resulting from lipid peroxidation [34]. Our study showed that acute CCl_4_ treatment caused an increase of liver MDA concentration which was in agreement with reported result [35]. Pretreatment with MF at the doses of 66, 132, 264 mg/kg for four consecutive days reversed these changes. SOD which is an important in vivo antioxidant enzyme is inactivated by lipid peroxides or reactive oxygen species when CCl_4_ is administrated [36]. In the MF treated groups, a tendency of dose-dependent-increase of SOD activities was observed, with the effect of 264 mg · kg^−1^ being highly significant (*P* < 0.01). The strong DPPH scavenging activity of MF, and the increased liver SOD and decreased liver MDA levels of MF treated groups suggest that the hepatoprotective effects of MF are partly due to its antioxidant effects.

Chemical analysis revealed that MF contained large amounts of triterpene compound-ursolic acid, as well as phenolic compounds-ellagic acid and flavonoids (glycosides of apigenin, luteolin and quercetin). All these natural products are known to have hepatoprotective activities [37–39]. The active fraction of Gurigumu 7, MF, with better antioxidant and hepatoprotective activity in a much less dosage than Gurigumu 7, could be a better choice for patients.

## Conclusions

In conclusion, this study demonstrated for the first time that the methanol eluted fraction (MF) from a DIAION column of an extract of the Mongolian medicinal prescription-Gurigumu-7 has hepatoprotective effect. MF dose dependently decreased serum aminotransferase activities, increased liver SOD levels and decreased liver malonyldialdehyde levels in CCl_4_ treated mice. MF was found to contain large amounts of bioactive phenolic compounds and triterpenes which might act concertedly for the hepatoprotective effects. The research results provided scientific evidence for the clinic efficacy of this Mongolian medicinal prescription for liver diseases. The results demonstrated that MF is a promising bioactive fraction of Gurigumu-7 for the develpment of convenient hepatoprotective formulations.

## Abbreviations

A, absorbance; ALT, alanine transaminase; AST, aspartate transaminase; EC_50_, 50 % effective concentration; MDA, malonyldialdehyde; MF, the methanol eluted fraction from a DIAION column of an extract of Gurigumu-7; MRM, multiple reaction monitoring; SOD, superoxide dismutase; UPLC-DAD-QQQMS, ultra-high performance liquid chromatography-diode array detector-triple quadruple mass spectrometry
